# Effects of the Increase in Employment of Women

**Published:** 1915-11-20

**Authors:** 


### NATIONAL INSURANCE ACT DEVELOPMENTS.

### EFFECTS OF THE INCREASE IN EMPLOYMENT OF WOMEN.

The heavy demands which the war has already made upon industrial labour have not only resulted in the employment of women in occupations hitherto exclusively confined to men—such, for example, as tram-conductors and railway-ticket collectors, but have also required the employment of women in far greater numbers in those occupations where female labour had already come to be recognised—as, for instance, in the case of clerks. The war has, in fact, brought into existence not only a vast fighting army, but a new army of industrial workers. The latter are, most of them, brought for the first time under the provisions of the National Insurance Acts as employed persons, and their enrolment as members of approved societies has proceeded and is proceeding apace. Statistics of the numbers who have thus entered insurance for the first time are, so far as we know, non-existent, and as the numbers are growing day by day it is not certain that such statistics, at the present moment, would be of much value if obtained. Common observation, however, is sufficient to show that the vast majority of these fresh levies to the industrial army are not entering insurance at the age of sixteen years, and it therefore follows that the sickness benefit to which most of these new insurance entrants are entitled is less than the rate which has come to be considered the normal for women—namely, 7s. 6d. a week.

This unexpected development of the labour market, on such a widespread scale, raises several interesting and important questions.

### Will the Sickness Rate be Reduced?

In the first place, if it be true that the normal sickness rate among women is greater than that assumed in the original estimates, so that, in effect, the benefits are larger than the amount which the joint contributions of the employee and employer, as subsidised by the State, will produce, it seems at first sight that the large influx of women workers must occasion some uneasiness as to the burden thereby placed upon the finances of National Insurance, and particularly with regard to those approved societies which cater especially for female industrial workers. It must, however, be borne in mind that one of the principal causes assigned in their report by the committee appointed some eighteen months or two years ago to investigate excess sickness claims on approved societies for the high rate of sickness claim in women's societies was the low rate of wages paid for female labour. It was held that the low rate of wages had a detrimental effect in at least two directions. On the one hand the small amount available for expenditure upon food and other necessaries led to improper nourishment, and consequently to decreased power to resist disease and sickness; while, on the other hand, the receipt of 7s. 6d. a week during illness from national insurance funds, if it did not actually exceed the ordinary wage, at least bore a higher ratio thereto than the 10s. a week benefit to the normal wage in the case of a man.

Now it is a remarkable fact that the large influx of women into the ranks of employed contributors has coincided with a large increase in the rate of wages paid, and although to some extent the effectiveness of this increase may have been neutralised by the rise in prices of food and commodities generally, yet there is little reason to doubt that women workers are now in a better position than ever before to provide themselves with proper nourishment. Thus it may well happen that the rate of claim for sickness benefit will actually decrease for the class of women who are normally employed.

It is doubtless true that the work now being done is more strenuous than in normal times, both in intensity and as regards the number of hours a week spent at the factory or workshop, but the vast army of female workers are keenly alive to the urgency and importance of the work upon which they are engaged, and there is no temptation to the slackers to absent themselves from work on account of trivial ailments, which formerly might have been a sufficient excuse for putting forward a claim for sickness benefit. Moreover, as regards the new entrants into insurance, it is probably true that to a large extent they have been recruited from those whose means enable them to live in more healthy surroundings than those usually associated with female industrial labour. The new entrants may, in fact, be subject to entirely different experience in regard to sickness from those who until now have formed the majority of female industrial workers.

Thus, while there are those who look upon the unexpected influx of so large a number of women into insurance as a source of uneasiness from the financial point of view, we think that there are other elements in the situation which may have a vastly important influence in reducing the rate of claim for benefit, and from the point of view of immediate conditions we should not be surprised to find that the larger demand upon female labour is to women's approved societies a blessing in disguise.

### A Problem for the Future.

But the problem is not one which has reference only to the immediate present. There must necessarily arise some serious aspects of the question which will have to receive attention in the future, and which should be given some consideration even now. For instance, it can hardly be supposed that upon the cessation of hostilities there will be anything like the same demand for female labour as at the present time. The services of those engaged in munition work, for example, will be dispensed with almost immediately upon the conclusion of peace, while women who are engaged in various temporary employments will quickly find that, as the men return who have been promised that their places shall be kept open for them, they will be no longer required in the same business house which has furnished them with employment during the war. In some directions it is possible that the advent of female labour as a temporary expedient will have shown an employer how he can reduce expenses or make use of female hands to advantage permanently; but the British employer, on the whole, is so conservative that it is not to be anticipated that there will be any violent change in the normal composition of staff at the conclusion of the war.

Furthermore, many of the women now employed in factories and workshops will be required to take their accustomed places in domestic affairs. Thus on all hands it is clear that the country must be prepared for a return to conditions approaching the pre-war standard in the labour market in due course. This opens up the question as to what is to happen to the insurances thus begun. Are they to lapse, and the benefit of contributions already paid be lost? Or is any provision to be made for giving due value for the contributions paid in these exceptional circumstances? The problem is not, of course, a new one. Under the National Insurance scheme a person passes automatically out of insurance, unless he be engaged in manual labour, when his rate of remuneration exceeds £160 a year, and consequently there are many who in normal times pay insurance contributions for a period and pass out of insurance without receiving any actual benefit. In similar fashion, those who cease to be employed within the meaning of the Act lose the right to continue in insurance, except in certain circumstances as voluntary contributors.

But although the problem is not new, the extent to which the return to uninsured employment will take place after the war is likely to be so large that some special steps will have to be taken in justice to those who have taken an honourable part in the work of the nation in war-time.

